# Isolation, genetic characterization, and virulence profiling of different *Aeromonas* species recovered from moribund hybrid catfish (*Clarias* spp.)

**DOI:** 10.14202/vetworld.2023.1974-1984

**Published:** 2023-09-23

**Authors:** Dini Siswani Mulia, Rarastoeti Pratiwi, Widya Asmara, Mohamad Azzam-Sayuti, Ina Salwany Md. Yasin, Alim Isnansetyo

**Affiliations:** 1 Department of Biology Education, Faculty of Teacher Training and Education, Universitas Muhammadiyah Purwokerto, Jl. K.H. Ahmad Dahlan, Purwokerto 53182, Indonesia; 2 Department of Biology, Faculty of Biology, Universitas Gadjah Mada, Jl. Teknika Selatan, Senolowo, Yogyakarta 55281, Indonesia; 3 Department of Microbiology, Faculty of Veterinary Medicine, Universitas Gadjah Mada, Jl. Fauna, Caturtunggal, Yogyakarta 55281, Indonesia; 4 Laboratory of Aquatic Animal Health and Therapeutics, Institute of Bioscience, Universiti Putra Malaysia, 43400 Serdang, Selangor, Malaysia; 5 Department of Fisheries, Faculty of Agriculture, Universitas Gadjah Mada, Jl. Flora, Bulaksumur, Yogyakarta 55281, Indonesia

**Keywords:** 16S rDNA, aeromoniasis, motile *Aeromonas* septicemia, pathogenicity, phenotype, phylogenetic

## Abstract

**Background and Aim:**

The high diversity of *Aeromonas* spp. results in various pathogenicity levels. This group of bacteria causes a serious disease named motile *Aeromonas* septicemia (MAS) in catfish (*Clarias* spp.). This study aimed to characterize the species and virulence gene diversity of *Aeromonas* spp. isolated from diseased catfish.

**Materials and Methods:**

Nine *Aeromonas* spp. were isolated from infected catfish cultivated in Java, Indonesia, and they were identified at the phenotypic and molecular levels (16S rDNA). The virulence genes assessed included *aer/haem*, *alt*, *ast*, *flaA*, *lafA*, and *fstA*.

**Results:**

Phylogenetic analysis identified nine isolates of *Aeromonas* spp.: *Aeromonas hydrophila* (11.11%), *Aeromonas caviae* (11.11%), *Aeromonas veronii* bv. *veronii* (44.44%), and *Aeromonas dhakensis* (33.33%). Virulence genes, such as *aer/haem*, *alt*, *ast*, *flaA*, *lafA*, and *fstA*, were detected in all isolates at frequencies of approximately 100%, 66.67%, 88.89%, 100%, 55.56%, and 66.67%, respectively. This study is the first report on *A. dhakensis* recovered from an Indonesian catfish culture. Furthermore, our study revealed the presence of *A*. *veronii* bv *veronii*, a biovar that has not been reported before in Indonesia.

**Conclusion:**

This finding confirms that MAS was caused by multiple species of *Aeromonas*, notably *A. dhakensis* and *A*. *veronii* bv *veronii*, within Indonesian fish culture. The presence of these *Aeromonas* species with multiple virulence genes poses a significant threat to the freshwater aquaculture industry.

## Introduction

Catfish (*Clarias* spp.) is a freshwater aquaculture species with global potential. However, the disease caused by *Aeromonas* spp. is one of the most serious and devastating causes of losses in catfish culture [1–3]. These Gram-negative, oxidase-positive, and facultative anaerobic, bacteria normally live in an aquatic environment [4–6]. *Aeromonas* spp. causes considerable losses to the aquaculture industry and ubiquitous pathogens. Several *Aeromonas* spp. can infect marine fish, other organisms [7–10], and humans [11].

Most *Aeromonas* spp. cause aeromoniasis or motile *Aeromonas* septicemia (MAS), which features specific clinical signs of erosion and hemorrhage in the mouth and on the body surface, erosion, hyperemia, congestion, abscess, necrosis, dropsy, abdominal dropsy, abdominal ascites, and exophthalmia [12–18]. Infection by *Aeromonas* spp. also causes histological changes in the organs of the affected fish. Such conditions result in decreased levels of hemocyte aggregation in the digestive system of infected fish, massive aggregation of hemocytes and pyknotic nuclei in the hepatopancreas, and hemocyte aggregation and necrotic cells in gills [19]. In humans, *Aeromonas* spp. infect skin, soft tissues, and gastrointestinal tract and causes hepatobiliary disease, diarrhea, and bacteremia [11, 20, 21].

Twenty-four species of the *Aeromonas* complex had been identified as of 2010 [22]. By 2017, this number had grown to 32 [23]. The *Aeromonas* complex includes 36 species as reported in 2020 [24], which indicates the possibility of further expansion of this genus.

Based on biochemical characterization and 16S rDNA sequences, the main etiologic agents for aeromoniasis in eels in Europe, Japan, and Korea include *Aeromonas hydrophila*, *Aeromonas veronii*, *Aeromonas sobria*, *Aeromonas caviae*, *Aeromonas jandaei*, *Aeromonas aquariorum*, *Aeromonas* *media*, and *Aeromonas trota* [25–27]. These species may also infect other aquaculture species, including catfish. In addition to its high species diversity, most members of the genus *Aeromonas* include are pathogenic. Their pathogenicity is related to their virulence factors. The complex virulence of *Aeromonas* features is caused by some factors contributing to the development of the infection process [28]. The virulence is strictly related to the expression of genes recognized in *Aeromonas* spp. Extracellular products, such as hemolysin, protease and lipase, toxin, and structural components (flagella, fimbriae, lipopolysaccharide, and outer-membrane protein), whether acting together or individually, enable the microorganisms to colonize and infect the host [29]. Several virulence genes have been identified to play a role in the pathogenicity of *Aeromonas* spp. [30].

The total catfish production in Indonesia reached 1.06 million tons in 2021 and 1.03 million tons in 2020, which indicate a production increment of 2.8% [31]. Disease outbreaks have resulted in a low increment in total fish production in Indonesia in 2020–2021. The previous reports by Mulia *et al*. [32], Marnis and Iswanto [33], Sarjito *et al*. [34], Rejeki *et al*. [35], and Yuliantoro *et al*. [36] have described *Aeromonas* spp. as a causative disease agent in catfish. The *Aeromonas* species identified from fish in Indonesia include *A. hydrophila*, *Aeromonas salmonicida*, *Aeromonas sobria*, *A. caviae*, *A. media*, *Aeromonas taiwanensis*, and *A. jandaei* [37–39], while those from pond water comprise *A. hydrophila* and *A. veronii* [40]. Records of the diverse types of pathogenic *Aeromonas* are not yet available at the global level or in Indonesia. Therefore, a study on the etiological agents of catfish aeromoniasis is required. In addition, the identification of *Aeromonas* spp. based on phenotype characteristics along is very difficult and inaccurate. Thus, genotypic identification supported by phenotypic characters and virulence data is required.

This study aimed to characterize the species and virulence gene diversity of *Aeromonas* spp. isolated from diseased catfish.

## Materials and Methods

### Ethical approval

The management, conditions, and procedures of the experiment in this study were approved by the Ethical Clearance Commission of Universitas Gadjah Mada (approval # certificate: 00137/04/LPPTI/|/201).

### Study period and location

The study was conducted from May 2017 to January 2019. The samples were collected from aquaculture ponds in four provinces in the Special Region of Yogyakarta, Central Java, West Java, and East Java, Indonesia.

### Isolation and bacterial culture

Sixty-seven fish with a size of 3.5–30 cm was obtained. Bacteria were isolated from the kidneys of diseased catfish The bacteria were cultured in glutamate starch phenyl (GSP) medium (Merck, Darmstadt, Germany), a selective medium for *Aeromonas* and *Pseudomonas*, at 30°C for 24 h. *Aeromonas* and *Pseudomonas* grew in yellow and red colonies, respectively. Furthermore, yellow single colonies were recovered in the tryptic soy broth (TSB) medium (Merck). The isolates were stored in TSB medium (Merck) with 20% glycerol at −80°C for further assay.

### Phenotypic identification of *Aeromonas* spp.

Phenotypic identification was performed by observing the colonies, cell morphology, and biochemical properties of the bacteria. The bacteria were grown on tryptic soy agar (TSA) medium (Merck) at 30°C for 24 h. Biochemical characterizations included the oxidative/fermentative (F), Voges-Proskauer (VP) test, citrate utilization, lysine utilization, ornithine utilization, urease production, phenylalanine deamination, nitrate reduction, H_2_S production, glucose utilization and gas production, adonitol, lactose, arabinose, sorbitol utilization, and aesculin hydrolysis which were conducted using the biochemical identification kit (HiMedia, India).

### Bacterial genomic DNA extraction

A total of 1 mL of bacterial culture in TSB medium was incubated at 30°C for 24 h and centrifuged at 13,000× *g* for 2 min. The bacterial genomic DNA was extracted using a bacterial DNA kit (Promega, Madison, USA) in accordance with the manufacturer’s protocol. The extracted bacterial DNA was stored at −20°C for further assay.

### 16S rDNA amplification

The 16S rDNA was amplified using the universal oligonucleotide primers 27F and 1492R (Table-1) [31–35]. The total volume of 25 μL contained 12 μL Mytaq HS Red Mix (Bioline, Meridian Life Science, Memphis, UK), 2×polymerase chain reaction [PCR] Master Mix (Bioline, Meridian Life Science, Memphis, UK), 1 μL forward primer, 1 μL reverse primer, 1 μL DNA template (20 ng), and 10 μL nuclease-free water (NFW) [16]. The PCR cycling program was implemented as follows: An initial denaturation at 95°C for 3 min; 30 cycles of denaturation at 95°C for 30 s, annealing at 55°C for 30 s, and extension at 72°C for 90 s; and a final extension at 72°C for 5 min. The PCR products were separated by electrophoresis on a 1% agarose gel before sequencing (1^st^ BASE Laboratories, Selangor, Malaysia).

**Table-1:** Primer sets used in this study.

Gene	Gene product	Primer sequence (5’- 3’)	Product size (bp)	Annealing temperature (°C)	Reference
*16 S*	16 S rRNA gene	F: AGA GTT TGA TCM TGG CTC AG R: TAC GGY TAC CTT GTT ACG ACT T	1500	55	[31]
*aerA/haem*	Aerolysin/Hemolysin	F: CCT ATG GCC TGA GCG AGA AG R: CCA GTT CCA GTC CCA CCA CT	431	56	[32]
*Alt*	Heat-labile cytotonic enterotoxin	F: TGA CCC AGT CCT GGC ACG GC R: GGT GAT CGA TCA CCA CCA GC	442	56	[33]
*ast*	Heat-stabile cytotonic enterotoxin	F: TCT CCA ATG CTT CCC TTC ACT R: GTG TAG GGA TTG AAG AAG CCG	331	56	[33]
*flaA*	Polar flagellum	F: TCC AAC CGT YTG ACC TC R: GMY TGG TTG CGR ATG GT	608	56	[33]
*lafA*	Lateral flagellum	F: CCA ACT T (T/C) G C (C/T) T C (T/C) (C/A) TGA CC R: TCT TGG TCA T (G/A) T TGG TGC T (C/T)	736	50	[34]
*fstA*	Ferric siderophore receptor	F: CGC TCG CCC ATC CCC CTC TG R: GCC CCT TGC ACC CCC ACC ATT	452	55	[35]

### Detection of virulence genes

Virulence genes were amplified by PCR. The total PCR volume of 25 μL contained 12 μL Mytaq HS Red Mix (Bioline), 2× PCR Master Mix (Bioline), 1 μL forward primer, 1 μL reverse primer, 1 μL DNA sample (20 ng), and 10 μL NFW [16]. The virulence genes detected included *aerA/haem*, *alt*, *ast*, *flaA*, *lafA*, and *fstA* (Table-1). The PCR products were subjected to electrophoresis on a 1.5% agarose gel.

### Sequencing and phylogenetic analysis

Descriptive analysis was conducted on phenotypic identification and virulence gene detection results. The DNA sequences were edited and assembled using the DNA Baser program (Dacia/P7, Mioveni 115400, Arges, Romania, http://www.dnabaser.com/news/index.html) [41]. The degree of similarity was analyzed using the basic local alignment search tool (BLAST) program (http://www.ncbi.nlm.nih.gov/BLAST). The Clustal W program (Bio.tools organization, http://www.clustal.org/) was used for multiple sequence alignments. Phylogenetic trees with 1000 replications of bootstrap analysis were constructed using the maximum likelihood MEGA 7.0.26 package (The Pennsylvania State University, USA; https://www.megasoftware.net/) [42].

## Results

### Clinical signs of diseased catfish

**Figure-1:**
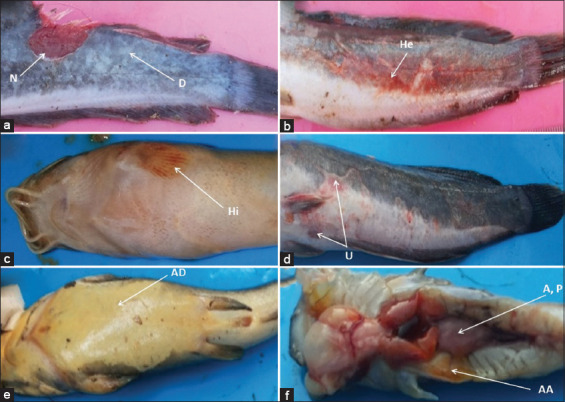
Clinical signs of moribund hybrid catfish diseased. a: Depigmentation (D) of the skin, necrosis (N), b: Hemorrhagic (He), c: Hyperemia (Hi), d: Ulcer (U), e: Abdominal dropsy (AD), abdominal ascites (AA), f: Abscess (S), pale (P) in kidney, abdominal ascites (AA).

Figure-1 shows the clinical signs of diseased catfish. The clinical signs observed included skin depigmentation, necrosis, hemorrhagic, hyperemia, ulcer, abdominal dropsy, abdominal ascites, abscess, and paleness in the kidney.

### Phenotypic characteristics of *Aeromonas* spp.

Morphological and biochemical characteristics suggested that the isolates observed in this study were members of *Aeromonas*. These bacteria grew well on GSP and TSA media at 30°C. They were rod-shaped, Gram-negative, motile, and F and can reduce nitrate (100%). These bacteria can also decarboxylate ornithine, deaminate phenylalanine, and utilize adonitol in the range of 11%–89% (v), but cannot produce acetyl methyl carbinol (VP), urease, and H_2_S. Two isolates of *Aeromonas* spp. (22.22%) can decarboxylate lysine, and seven isolates (77.78%) can decarboxylate lysine in the range of 11%–89% (v). However, only one isolate can utilize citrate (11.11%). Seven isolates (77.78%) were acid-forming (from glucose), with two isolates (22.22%) forming acids (from glucose) in the range of 11%–89% (v). One isolate (11.11%) produced gas from glucose. Five isolates (55.56%) utilized lactose, and four isolates (44.44%) used lactose in the range of 11%–89% (v). Eight isolates (88.89%) can utilize arabinose, and one isolate (11.11%) can utilize arabinose in the range of 11%–89% (v). Four isolates (44.44%) utilized sorbitol, and one isolate (11.11%) did not. Four isolates (44.44%) used sorbitol in the range of 11%–89% (v). Three isolates of *Aeromonas* spp. (33.33%) hydrolyzed aesculin (Table-2).

**Table-2:** The phenotypic characters of *Aeromonas* spp. bacteria isolated from diseased catfish.

Characterization	Isolates								

	Ah-01	Ac-01	Av-01	Av-02	Av-03	Av-04	Ad-01	Ad-02	Ad-03
Colony morphology									
Form	Circular	Circular	Circular	Circular	Circular	Circular	Circular	Circular	Circular
Edge	Even	Even	Even	Even	Even	Even	Even	Even	Even
Elevation	Convex	Convex	Convex	Convex	Convex	Convex	Convex	Convex	Convex
Color in TSA	White	White	White	White	White	White	White	White	White
Color in GSP	Yellow	Yellow	Yellow	Yellow	Yellow	Yellow	Yellow	Yellow	Yellow
Bacterial morphology									
Form	Rod	Rod	Rod	Rod	Rod	Rod	Rod	Rod	Rod
Gram	-	-	-	-	-	-	-	-	-
Motility	+	+	+	+	+	+	+	+	+
O/F	F	F	F	F	F	F	F	F	F
Voges-Proskauer	-	-	-	-	-	-	-	-	-
Citrate utilization	-	-	-	-	-	-	-	+	-
Lysine decarboxyzation	v	v	v	v	+	v	v	v	+
Ornithine decarboxyzation	v	v	v	v	v	v	v	v	v
Urease	-	-	-	-	-	-	-	-	-
Phenylalanine deamination	v	v	v	v	v	v	v	v	v
Nitrate reduction	+	+	+	+	+	+	+	+	+
H_2_S production	-	-	-	-	-	-	-	-	-
Glucose, acid	+	+	v	+	+	+	v	+	+
Glucose, gas	-	-	-	-	+	-	-	-	-
Adonitol	v	v	v	v	v	v	v	v	v
Lactose	+	v	+	v	+	v	v	+	+
Arabinose	+	+	+	+	+	v	+	+	+
Sorbitol	v	v	v	v	+	-	+	+	+
Aesculin hydrolysis	-	+	-	-	-	-	+	-	+

+, >90% of strains positive; –, >90% of strains negative; v, 11%–89% of strains positive; F=Fermentative, TSA=Tryptic soy agar, GSP=Glutamate starch phenyl, O/F=Oxidative/Fermentative

### Molecular identification of *Aeromonas* spp. based on 16S rDNA

**Figure-2:**
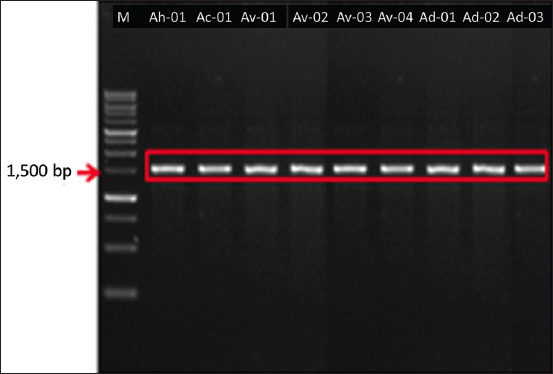
Results of amplification of bacterial DNA isolated with 16S rDNA primers. Arrow: 1500 bp, Marker: 1 kb.

The results of 16S rDNA amplification for nine isolates revealed base lengths of approximately 1500 bp (Figure-2). The 16S rDNA sequences of these isolates were compared with those of 28 isolates from the GenBank database (NCBI, USA). The homology search based on the 16S rDNA sequence (1410–1479 bp) showed that the nine isolates demonstrated similarities to *A. hydrophila*, *A. caviae*, *A. veronii* bv *veronii*, and *Aeromonas dhakensis*. Queries reached up to 96%–100% with a similarity range of 97%–99.86% (Table-3).

**Table-3:** Identity of *Aeromonas* spp. bacteria isolated from diseased catfish.

Isolates	Closest comparator	Query (%)	Identity (%)	Accession number
Ah-01	*Aeromonas hydrophila* ATCC 7966	99	99.45	NR118944
Ac-01	*Aeromonas caviae* ATCC 15468	97	99.57	NR029252
Av-01	*Aeromonas veronii* bv*. veronii* ATCC 35624	96	99.51	NR118947
Av-02	*Aeromonas veronii* bv*. veronii* ATCC 35624	100	99.72	NR118947
Av-03	*Aeromonas veronii* bv*.* *veronii* ATCC 35624	99	98.55	NR118947
Av-04	*Aeromonas veronii* bv*. veronii* ATCC 35624	96	99.72	NR118947
Ad-01	*Aeromonas dhakensis* P21	99	97.91	NR042155
Ad-02	*Aeromonas dhakensis* P21	100	99.86	NR042155
Ad-03	*Aeromonas dhakensis* P21	100	99.86	NR042155

Isolate Ah-01 was closest to *A. hydrophila* ATCC 7966 (NR118944) [43], with a similarity rate of 99.45% and query match of 99%. Isolate Ac-01 was closest to *A. caviae* ATCC 15468 (NR029252), with a similarity rate of 99.57% and 97% query matching. The Av-01, Av-02, Av-03, and Av-04 isolates were closest to *A. veronii* bv *veronii* ATCC 35624 (NR118947) [44]. The levels of similarity of these isolates were in the range of 98.55%–99.72% with 96%–100% query matching. Three isolates (33.33%), namely, Ad-01, Ad-02, and Ad-03 were the most closely related to *A. dhakensis* strain P21 (NR042155) [45]. Their levels of similarity were 97.91%–99.37% with 99%–100% query matching.

### Phylogenetic tree

**Figure-3:**
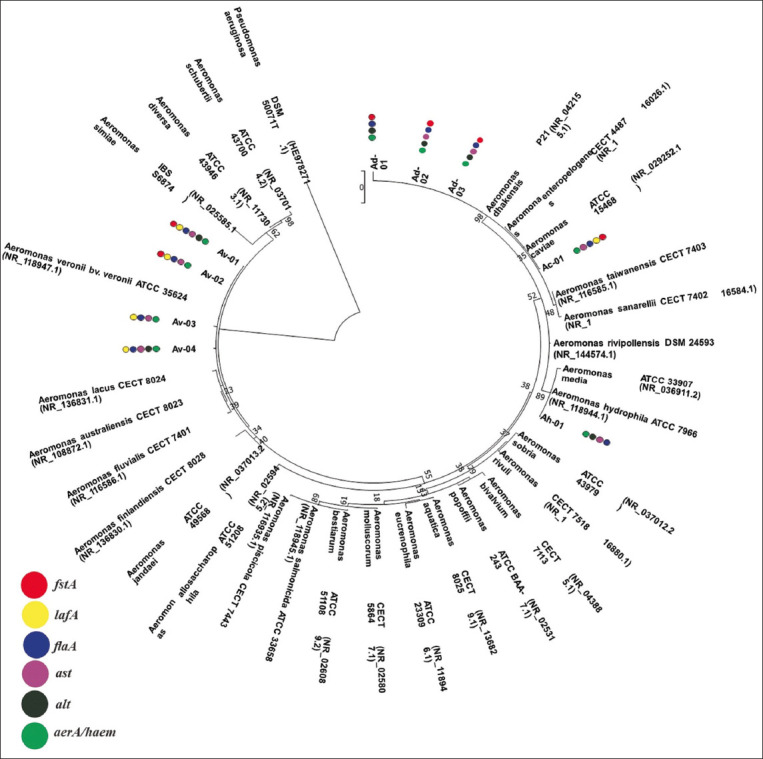
Phylogenetic tree constructed from the 16S rDNA sequences of *Aeromonas* sp. and other *Aeromonas* species (class of Gammaproteobacteria) and detected virulence genes. *Pseudomonas aeruginosa* was used as an outgroup. The topology was obtained by maximum likelihood with bootstraps of 1000 replications. The scale bar signifies 0.02 substitutions per nucleotide position (Knuc). Colored circles indicate the presence of the virulence genes for each isolate, 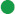 *aer/haem*: aerolysin/hemolysin; 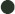 *alt*: heat-labile cytotonic enterotoxin; 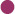 *ast*: heat-stable cytotonic enterotoxin; 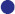 *flaA*: polar flagellum; 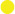 *lafA*: lateral flagellum; 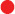 *fstA*: ferric siderophore receptor.

Phylogenetic analyses showed that Ah-01 and Ac-01 isolates were included in the clade of *A. hydrophila* ATCC 7966 and *A. caviae* ATCC 15468, respectively. Av-01, Av-02, Av-03, and Av-04 isolates were grouped into the clade of *A*. *veronii* bv *veronii* ATCC 35624 and Ad-01, Ad-02, and Ad-03 isolates into the clade of *A. dhakensis* P21 (Figure-3).

### Detection of virulence genes

Six virulence genes (*aer/haem*, *alt*, *ast*, *flaA*, *lafA*, and *fstA*) of the *Aeromonas* spp. were detected (Figure-3 and Table-4). The *aer/haem* gene was found in all isolates, and the *alt* gene was present in six isolates. The virulence gene *ast* was discovered in eight isolates, and *flaA* was amplified in all isolates. The virulent gene *lafA* was found in five isolates, and *fstA* was found in six isolates.

**Table-4:** Virulence genes in the *Aeromonas* spp. isolated from catfish.

Isolates	Species	The presence of virulence genes						Detection rate (%)

		*aerA/haem*	*alt*	*ast*	*flaA*	*lafA*	*fstA*	
Ah-01	*Aeromonas hydrophila* strain ATCC 7966	+	+	+	+	-	-	4 (66.67)
Ac-01	*Aeromonas caviae* strain ATCC 15468	+	-	+	+	+	+	5 (83.33)
Av-01	*Aeromonas veronii* bv*. veronii* strain ATCC 35624	+	+	+	+	+	+	6 (100.00)
Av-02	*Aeromonas veronii* bv*. veronii* strain ATCC 35624	+	-	+	+	+	+	5 (83.33)
Av-03	*Aeromonas veronii* bv*.* *veronii* strain ATCC 35624	+	-	+	+	+	-	4 (66.67)
Av-04	*Aeromonas veronii* bv*. veronii* strain ATCC 35624	+	+	+	+	+	-	5 (83.33)
Ad-01	*Aeromonas dhakensis* strain P21	+	+	-	+	-	+	4 (66.67)
Ad-02	*Aeromonas dhakensis* strain P21	+	+	+	+	-	+	5 (83.33)
Ad-03	*Aeromonas dhakensis* strain P21	+	+	+	+	-	+	5 (83.33)
Total		9 (100%)	6 (66.67%)	8 (88.89%)	9 (100%)	5 (55.55%)	6 (66.67%)	10

The four isolates of *A. dhakensis* harbored *aer/haem*, *alt*, *flaA*, and *fstA* but not *lafA*. However, three isolates harbored *ast* (Table-4 and Figure-3). The *aer/haem*, *ast*, *flaA*, and *lafA* were detected in all isolates of *A. veronii* bv *veronii*. However, *alt* and *fstA* were detected in two isolates, whereas only *A. caviae* isolate had *aer/haem*, *ast*, *flaA*, *lafA*, and *fstA*, but not *alt*. The genes *aer/haem*, *alt*, *ast*, and *flaA* were detected in *A. hydrophila* but *lafA* and *fstA* were missing.

The genes *aer/haem*, *alt*, *ast*, and *flaA* were detected in *A. hydrophila*, but *lafA* and *fstA* were absent. *A. caviae* isolate contained the *aer/haem*, *ast*, *flaA*, *lafA*, and *fstA*, but not *alt*. The *aer/haem*, *ast*, *flaA*, and *lafA* were detected in all isolates of *A. veronii* bv *veronii*. However, *alt* and *fstA* were detected in two isolates. The three isolates of *A. dhakensis* harbored *aer/haem*, *alt*, *flaA*, and *fstA* but did not harbor the *lafA* gene. However, two isolates harbored *ast* (Table-4 and Figure-3).

The PCR assay of all nine isolates detected the *aer/haem*, *alt*, *ast*, *flaA*, *lafA*, and *fstA* at the rates of 100%, 66.67%, 88.89%, 100%, 55.56%, and 66.67%, respectively. The results showed that all isolates had four or more virulence genes, and only one isolate (*A. veronii* bv *veronii* Av-01) had all six virulence genes.

## Discussion

This study investigated the diversity of *Aeromonas* spp. causing MAS in Indonesia and infected catfish. Specifically, this study aimed to determine the biodiversity of *Aeromonas* spp. in diseased catfish cultivated in four provinces on Java Island, Indonesia.

The clinical signs of diseased catfish included depigmentation of the skin, necrosis, hemorrhagic, hyperemia, ulcer, abdominal dropsy, abdominal ascites, abscess, and paleness of the kidney (Figure-1). Similar findings have been reported by Austin and Austin [46] and Dias *et al*. [47]. *Aeromonas* spp. can break the blood vessels, which results in ulcerative lesions with a hemorrhagic appearance throughout the tegument [48]. In this study, clinical signs of diseased catfish indicated typical signs of aeromoniasis. This finding suggests that the isolates of *Aeromonas* spp. considered in this study were pathogenic and infectious with remarkable clinical signs.

Based on phenotypic characteristics, this study identified nine isolates as presumptive *Aeromonas* species. The isolates were characterized based on their growth on GSP selective agar medium: yellow colonies, rod shape, Gram-negative, motile, and production of acid from glucose (Table-2). These characteristics have also been reported previously by El-Sharaby *et al*. [5]. In addition, *Aeromonas* can ferment glucose. A common characteristic of *Aeromonas* is their ability to produce gases from glucose. However, this study found only one isolate with such a trait, which suggests the inconsistency of this ability among *Aeromonas* [49]. In the present study, all *Aeromonas* isolates reduced nitrate, as previously reported by Vega-Sanchez *et al*. [50] for *A. caviae*, *A. veronii* bv *veronii*, and *A. hydrophila*. However, *Aeromonas* cannot produce acetyl methyl carbinol (VP), urease, and H_2_S. The previous study reported by Khor *et al*. [51] have also reported that all *Aeromonas* isolates are unable to produce urease and H_2_S. The ability of *Aeromonas* to produce acetyl methyl carbinol (VP) varies. *Aeromonas* *hydrophila* and *A. veronii* bv *veronii* manufacture this substance [52], but *A. caviae* does not [53]. The previous study by Khor *et al*. [51] have also revealed that *Aeromonas* varies in their ability to use citrate. This research found characteristics that are different from those of previous study by Borty *et al*. [52], revealing that *A. hydrophila* cannot utilize lysine; in addition, some species cannot utilize ornithine [51], but *A. veronii* bv *veronii* cannot specifically use arabinose [50, 54, 55].

In contrast to the findings of previous studies, *A. hydrophila* and *A. veronii* bv *veronii* cannot utilize sorbitol [50, 52], and hydrolyze aesculin [53], but can hydrolyze aesculin [50, 52, 54, 55]; meanwhile, 50% of *A. caviae* can hydrolyze aesculin [52]. These results indicate variations in the phenotypic characteristics of *Aeromonas*. This condition has also been reported previously by Liu [49] and Martin-Carnahan and Joseph [56]. Phenotypic variation is thought to have clinical significance for determining the relationship between biotype and enterotoxin production [53].

The identification of members of *Aeromonas* spp. at the species level remains a huge challenge given their genetic heterogeneity. Biochemical characteristics are ineffective for the identification of *Aeromonas* at the species level [57]; thus, species identification should be supported by genotypic analysis. Molecular identification using 16S rDNA is very helpful for accurate species identification [58, 59]. Furthermore, we proved the biodiversity of *Aeromonas* spp. by 16S rDNA sequencing. We used 16S rDNA sequence rather than *gyrB* sequence for molecular identification in this study. We performed preliminary screening for *Aeromonas* spp. using GSP medium based on its morphological characteristics, further confirmed using a series of biochemical tests that highly aligned with *Aeromonas* characteristics, and finally, 16S rDNA phylogenetical analysis with the inclusion of *Aeromonas* type strains. Results from our phylogenetic tree also revealed that our isolates formed a tight clade with their corresponding type strains. In addition, the numbers of 16S rRNA gene database in NCBI are much higher available than that of *gyrB* gene sequence. Based on the availability of the database, the initial molecular identification is likely more appropriate using the 16S rDNA sequence to avoid the mismatch results of similarity analysis.

The results of 16S rDNA amplification of nine isolates showed a base length of approximately 1500 bp (Figure-2). BLAST search of the 16S rDNA *Aeromonas* spp. revealed a high level of similarity, namely, 97.91%–99.86%, and queries reaching 96%–100% (Table-3). Homology searches conducted on the 16S rDNA sequence indicated that the nine isolates obtained were parallel to *A. hydrophila*, *A. caviae*, *A. veronii* bv *veronii*, and *A. dhakensis*. Our results indicate the successful identification of nine isolates of, *Aeromonas* species, namely, *A. hydrophila* (11.11%), *A. caviae* (11.11%), *A. veronii* bv *veronii* (44.44%), and *A. dhakensis* (33.33%). These results were confirmed using phylogenetic relationship analysis (Figure-3). The current findings completed previous results that identified three species, *A. hydrophila* [33], *A. caviae*, and *A. veronii* [34], from catfish in Indonesia during genotypic analysis. Indonesian researchers have previously identified *Aeromonas* species from catfish by phenotypic identification and collected three *Aeromonas* species, namely, *A. hydrophila*, *A*. *caviae*, and *A. salmonicida* [35, 60, 61]. Six species, including *A. hydrophila*, *A. salmonicida*, *A*. *veronii*, *A. media*, *A. taiwanensis*, and *A. jandaei* were isolated form other freshwater and pond fishes, and successfully identified genotypically [37, 39].

The previous study has also identified *Aeromonas* spp. from catfish (*Clarias gariepinus*) in Egypt using genotypic analysis, these bacteria consisted of *A. hydrophila* (56%) and *A. veronii* (44%) [62], *A. hydrophila* (50.4%), and *A. caviae* (5.6%) according to phenotypic analysis [63]. Other research on catfish in Uganda discovered *A. hydrophila* (33%) and *A. sobria* (33%) through genotypic analysis [13]. In the present study, 16S rDNA molecular identification in catfish revealed the high biodiversity of *Aeromonas* spp., that is, four species, compared with a previous study, which detected one or two *Aeromonas* species. The high biodiversity of *Aeromonas* spp. is presumably due to the large sampled locations, which covered three provinces in Java Island: DIY Yogyakarta, Central Java, and West Java. The former surveys only involved West Java of Indonesia [33], center Java of Indonesia [34], and Qena in Egypt [62], as well as the central region and northern region of Uganda [13]. In addition, the differences in type and number of *Aeromonas* species identified from catfish may be due to the differences in individual fish, cultivation systems, climates, and environmental influences.

In line with this study, *A. hydrophila*, *A. veronii*, and *A. caviae* have frequently been isolated from diseased catfish. Unexpectedly, in this study, the prevalence of *A. dhakensis* was observed. Although *A. dhakensis* is rarely found in fish, this species is virulent to Nile tilapia (*Oreochromis mossambicus*) in Mexico [64] and other fish in Australia [65]. In addition, *A. dhakensis* was first reported to cause acute hemorrhagic septicemia in experimentally infected pacu fish (*Piaractus mesopotamicus*) in Brazil [66]. *Aeromonas dhakensis* was the predominant *Aeromonas* species isolated from freshwater fish in nine fish farms in Malaysia [67]. *Aeromonas dhakensis* is a major emerging pathogen of striped catfish in Vietnam [68]. *Aeromonas dhakensis* is possibly an important pathogen in catfish and other freshwater fishes, either in Indonesia or around the world. In addition, *A. veronii* bv *veronii* is the first *A. veronii* biovar, which has been previously reported as *A. veronii*, reported in Indonesia [34, 39].

Numerous virulence genes associated with the pathogenicity of *Aeromonas* spp. act synergistically with each other to exhibit their pathogenicity. In this study, the biodiversity of the *Aeromonas* complex was due to variations in virulence genes. The pathogenicity of the *Aeromonas* complex is multifactorial and assumed to involve the products of some genes, such as aerolysin (*aer*) and hemolysin (*haem*) (extracellular products), two cytotoxic, heat-labile (*alt*) and heat-stable (*ast*) genes, polar flagellum (*fla*) (structural components), lateral flagellum (*laf*), and ferric siderophore receptor (*fst*) [22, 69]. To spread their virulence factors, *Aeromonas* employs four secretion systems that discharge cellular products into the extracellular environment or directly to the host cell [70].

To recognize the potential virulence of *Aeromonas* spp., scientists regard the detection of virulence genes as a practical method for screening a large number of *Aeromonas* isolates with a suitable virulence potential. The previous studies by Mulia *et al*. [16] and Pessoa *et al*. [29] have also reported the detection and characterization of virulence factors in *Aeromonas* spp. In the present study, six virulence genes, that is, *aer/haem*, *alt*, *ast*, *flaA*, *lafA*, and *fstA*, were detected (Table-4). This study also showed that *Aeromonas* have at least four virulence genes. Similar results have been reported for *aer/haem* and *flaA* [30, 62]. Aerolysin is a hemolytic extracellular product coded by the aerolysin gene and plays a significant role in the pathogenicity of *Aeromonas* spp. It is the most important toxin among potential virulence factors in *Aeromonas* [71]. *Aeromonas* has two types of flagella, that is, polar and lateral, which are coded by *flaA* and *lafA* genes, respectively [70]. *Alt* and *ast* have been detected in some *Aeromonas* spp. [65]. The present study detected *fstA* in some isolates of *Aeromonas* spp. species but it not in *A. hydrophila*. In contrast, other research on a variety of sources (seawater, water, diseased fish, bivalves, cake, and wound exudate) of *Aeromonas* detected *fstA* in *A*. *salmonicida*, but not in *A. hydrophila*, *A. caviae*, and *A. veronii* [70].

All findings of this study provide important information regarding the characteristics and species diversity of *Aeromonas*, virulence genes of *Aeromonas* spp., and the presence of *A. dhakensis* in catfish. *Aeromonas dhakensis* showed virulence potential due to some virulence genes it possesses, such as *aer/haem*, *alt*, *flaA*, and *fstA* genes (100%), as well as the *ast* gene (66.67%). Such information may be useful for determining strategies for the control of MAS disease in catfish and other freshwater fishes.

## Conclusion

Four species of *Aeromonas* spp., namely, *A*. *hydrophila*, *A. caviae*, *A. veronii* bv *veronii*, and *A. dhakensis* were identified in the current study. Virulence genes, including *aer/haem*, *alt*, *ast*, *flaA*, *lafA*, and *fstA* were detected at prevalence rates of 100%, 66.67%, 88.89%, 100%, 55.56%, and 66.67%, respectively. This study is the first report on the isolation of *A. dhakensis* from aquaculture species in Indonesia. Two isolates of *A. dhakensis* harbored five virulence genes, and one isolate possessed four virulence genes. In addition, the *A. veronii* bv *veronii* isolated in this study is the first *A. veronii* biovar isolated from aquaculture species in Indonesia. The isolates of *A. veronii* bv *veronii* possess six, five, or four virulence genes. This study has successfully revealed the diversity of *Aeromonas* spp. and their virulence genes. The current report contributes considerably to the basic consideration for the development of an *Aeromonas* vaccine.

## Authors’ Contributions

DSM: Conceived and designed, and performed the experiments, analyzed the data, prepared figures and/or tables, authored or revised the manuscript, and approved the final draft. RP and WA: Supervised the experiment and reviewed the manuscript. MA and ISMY: Analyzed and interpreted data and drafted and revised the manuscript. AI: Designed the experiments, proposed the grant proposal, contributed reagents/materials/analysis tools, and reviewed the manuscript. All authors read and approved the final manuscript.
